# Peripheral Giant Cell Granuloma in a Child Associated with Ectopic Eruption and Traumatic Habit with Control of Four Years

**DOI:** 10.1155/2016/6725913

**Published:** 2016-11-24

**Authors:** Luiz Evaristo Ricci Volpato, Cristhiane Almeida Leite, Brunna Haddad Anhesini, Jéssica Marques Gomes da Silva Aguilera, Álvaro Henrique Borges

**Affiliations:** ^1^Master's Program in Integrated Dental Sciences, University of Cuiabá, Cuiabá, MT, Brazil; ^2^Department of Oral Pathology, University of Cuiabá, Cuiabá, MT, Brazil; ^3^Master's Program in Restorative Dentistry, University of São Paulo, São Paulo, SP, Brazil; ^4^University of Cuiabá, Cuiabá, MT, Brazil

## Abstract

Peripheral giant cell granuloma (PGCG) is a nonneoplastic lesion that may affect any region of the gingiva or alveolar mucosa of edentulous and toothed areas, preferentially in the mandible and rarely occurring in children. This report describes the clinical and histopathological findings of a PGCG diagnosed in the maxilla of a 9-year-old boy associated with a tooth erupting improperly and a traumatic habit. The patient did not present anything noteworthy on extraoral physical examination or medical history, but the habit of picking his teeth and “poking” the gingiva. The oral lesion consisted of an asymptomatic, rounded, pink colored, smooth surface, soft tissue injury with fibrous consistency and approximated size of 1.5 cm located in the attached gingiva between the upper left permanent lateral incisor and the primary canine of the same side. Excisional biopsy was performed through curettage and removal of the periosteum, periodontal ligament, and curettage of the involved teeth with vestibular access. The histopathological analysis led to the diagnosis of PGCG. The prompt diagnosis and treatment of the PGCG resulted in a more conservative surgery and a reduced risk for tooth and bone loss and recurrence of the lesion. After four years of control, patient had no relapse of the lesion and good gingival and osseous health.

## 1. Introduction

The peripheral giant cell granuloma (PGCG) was first described in 1953 by Jaffe and was originally called reparative giant cell granuloma [1]. PGCG is a nonneoplastic lesion, characterized by reactive hyperplasia in the presence of local irritation, including trauma from extractions, food impaction, calculus, periodontal disease, periodontal surgery, orthodontic appliances, defective restorations with overhanging margins, and ill-fitting removable appliances [2–4]. They affect any region of the gingiva or alveolar mucosa of edentulous and toothed areas [2] and it is believed to be originated from periosteal or periodontal ligament cells [5, 6]. They can occur in different age groups, predominantly between the fourth and sixth decades of life [7, 8].

Clinically, the PGCG presents exophytic growth of sessile or pedicle base, reddish or purplish smooth surface, and consistency ranging from being soft to firm, with the mandible more often involved than the maxilla [2, 8, 9]. The clinical differential diagnosis of a reactive lesion of the gingiva must include pyogenic granuloma, traumatic fibroma, peripheral ossifying fibroma, and other lesions [10]. Early recognition, diagnosis, and treatment of this lesion are important. The treatment consists of local surgical excision below the underlying bone and removal of any irritation agent in the region in order to minimize the risk of relapse [10, 11].

This case report describes the clinical and histopathological findings of a PGCG diagnosed in the maxilla of a pediatric patient associated with a tooth erupting improperly and a traumatic habit along with four years of clinical and radiographic control.

## 2. Case Presentation

A 9-year-old boy was referred for treatment in the Pediatric Dentistry Clinic of the Cuiabá Dental School of the University of Cuiabá (UNIC) accompanied by his mother. The main complaint of the patient, reported by his mother, was the presence of a “ball of gingiva” with three months of progressive growth. There was nothing noteworthy at the extraoral physical examination. The medical history revealed no systemic diseases, and he was not in use of any medications at the time. Both the patient and his mother reported that he had the habit of picking his teeth and “poking” the gingiva.

**Figure 1:**
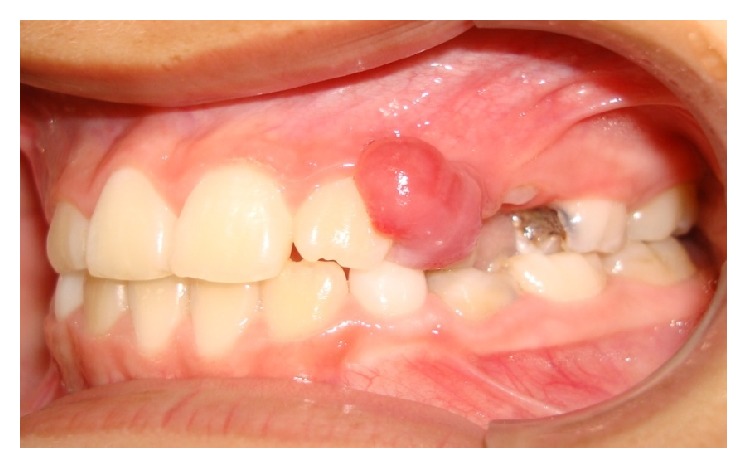
Nodular lesion between the upper left permanent lateral incisor and the primary canine.

The intraoral examination showed an asymptomatic, rounded, pink colored, smooth surface, soft tissue lesion. It had fibrous consistency, was resilient to the touch, and had the size of approximately 1.5 cm in its largest diameter, located in the attached gingiva between the upper left permanent lateral incisor and the primary canine of the same side (Figure 1). Patient was in mixed dentition with some active carious lesions and poor oral hygiene.

**Figure 2:**
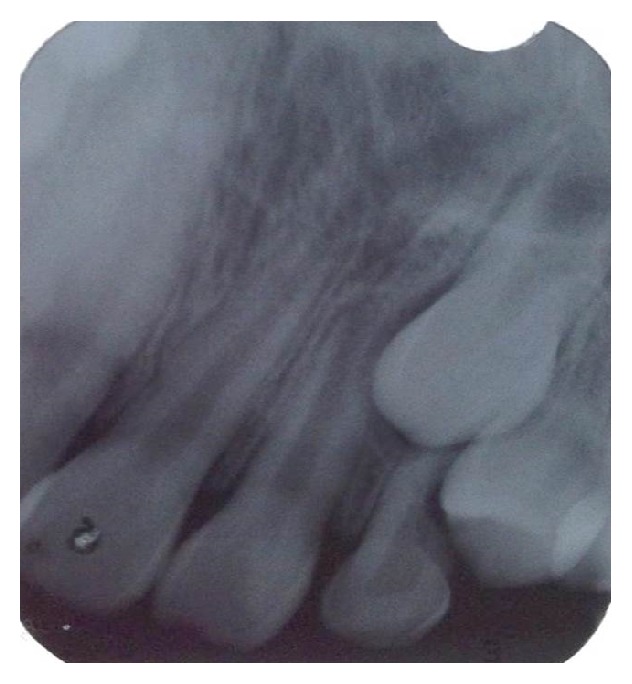
Radiographic aspect of the lesion without signs of abnormality.

No radiographic change was observed (Figure 2). Faced with clinical and radiographic findings, the presumptive diagnosis was pyogenic granuloma.

**Figure 3:**
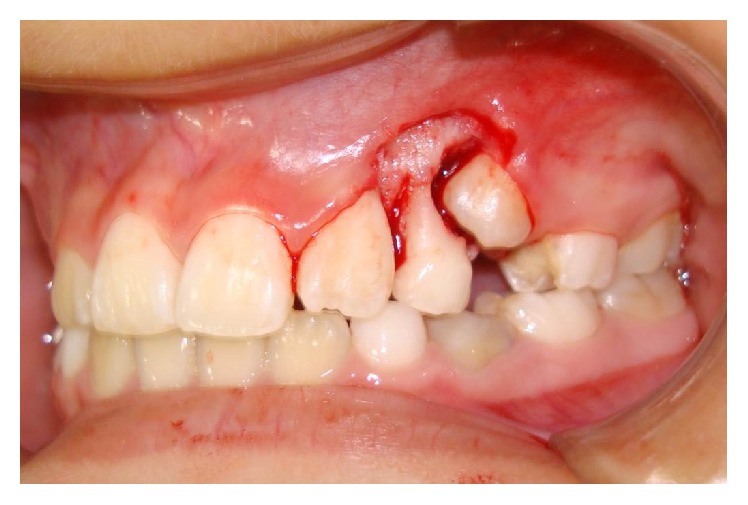
Clinical aspect after excisional biopsy of the lesion.

The patient was submitted to excisional biopsy of the lesion through curettage and removal of the periosteum, periodontal ligament, and curettage of the involved teeth with vestibular access. Surgical planning of the case included the preservation of the involved teeth which showed vitality and no increased mobility (Figure 3).

**Figure 4:**
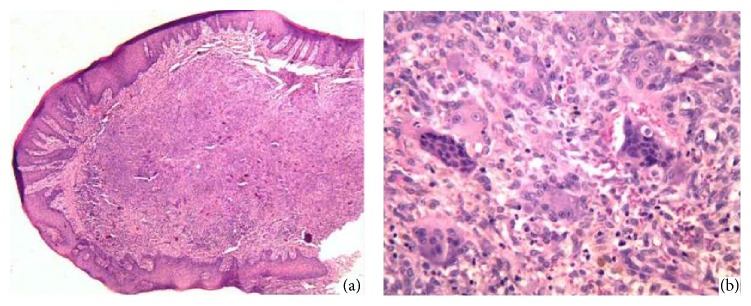
(a) Low magnification of PGCG. The stratified squamous epithelium exhibits hyperkeratosis and acanthosis. The subjacent fibrous connective tissue showed noncapsulated nodular proliferation of cellular mesenchymal tissue with abundant multinucleated giant cells dispersed throughout (H&E; original magnification ×20). (b) Higher magnification of PGCG showing giant cells, spindle-shaped ovoid plump stromal cells, inflammatory cells, capillaries, hemorrhage, and hemosiderophages (H&E; original magnification ×400).

Microscopic examination showed noncapsulated nodular proliferation of cellular mesenchymal tissue with abundant multinucleated giant cells dispersed throughout, surfaced by stratified squamous epithelium. Stromal cells consisted of spindle-shaped ovoid plump and mesenchymal cells. Mononuclear inflammatory cells, abundant capillaries, hemorrhage, and hemosiderophages were also observed. The histopathological diagnosis was peripheral giant cell granuloma (Figure 4).

**Figure 5:**
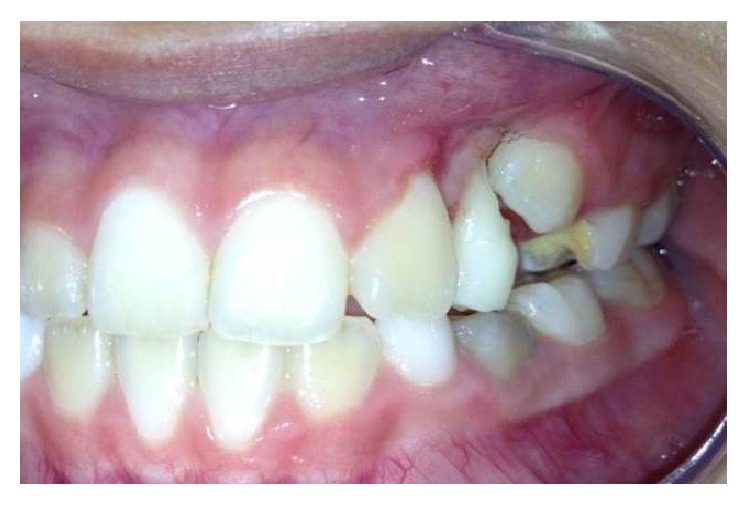
Seven-day control.

In the postoperative controls of 7 (Figure 5), 14, and 21 days, the area showed a good evolution, with the wound healing.

**Figure 6:**
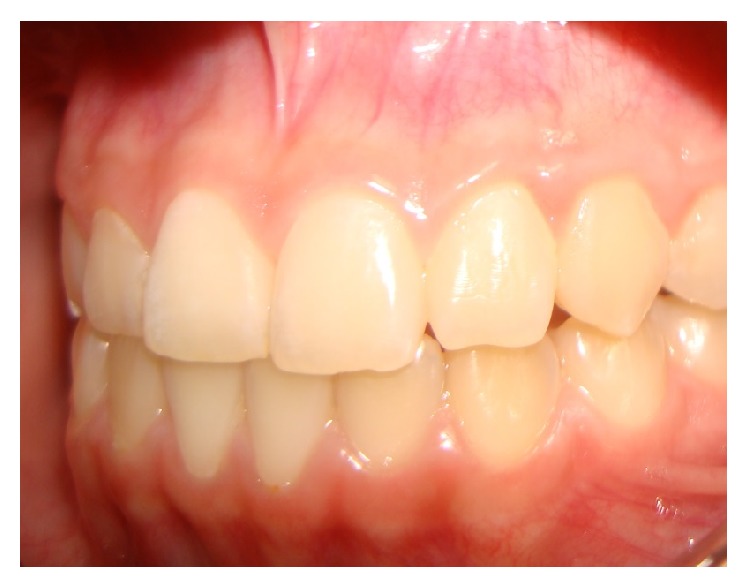
Clinical appearance after four years.

**Figure 7:**
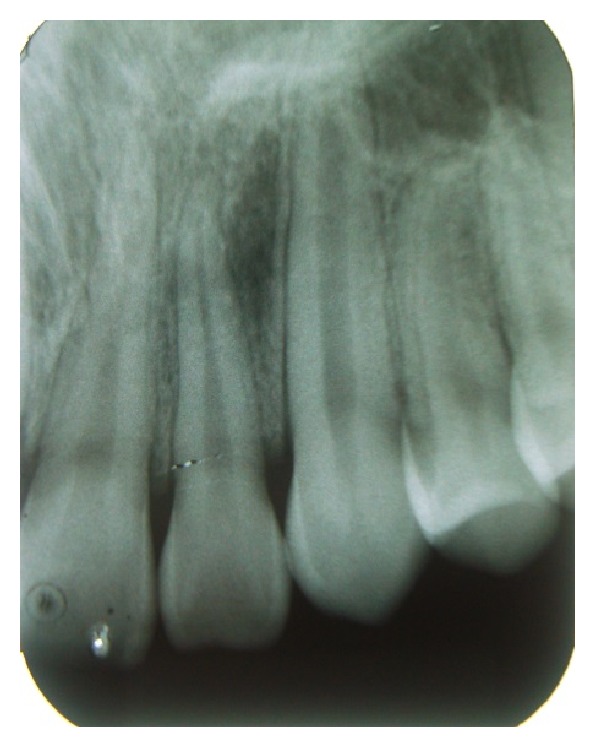
Radiographic control after four years without signs of abnormality.

After four years, the region shows no sign of relapse of the lesion and no clinical or radiographic alteration (Figures 6 and 7).

## 3. Discussion

The dentist is often faced with conditions involving inflammatory processes related to dental plaque, as gingivitis and periodontitis. However, some patients may have other pathological processes located in periodontium, such as PGCG [12]. The giant cell granuloma (GCG) is not a neoplasm, but a reactive lesion caused by trauma or irritation. Usually it occurs in patients with poor oral hygiene condition [13], as in the presented case. Only 9% of the cases occur in children aged up to 10 years and range from 6.5% to 12.7% in patients of 11–20 years [3, 11].

The origin of giant cells in PGCG is still unclear. Some authors concluded that the multinucleated cells in PGCG are of osteoclastic origin and are derived from differentiated mononuclear cells but the mechanism that activates or recruits osteoclasts in PGCG is still being investigated [14, 15].

In the presented case, the lesion was located in the interdental papilla and the initial clinical hypothesis was pyogenic granuloma (PG). Clinically PGCG, PG, peripheral ossifying fibroma (POF), and gingival fibromatosis (GF) are proliferative gingival lesions that can show very similar characteristics but can present distinct infiltrative features and recurrence risk [16]. They can be also easily distinguished from parulis, which is frequently associated with a necrotic tooth or with periodontal disorder [17]. PGCG like the peripheral ossifying fibroma is a lesion unique to the oral cavity, occurring only on the gingiva. Unlike peripheral ossifying fibroma, however, it may occur on the alveolar mucosa of edentulous areas. Like pyogenic granuloma and peripheral ossifying fibroma, peripheral giant cell granuloma may represent an unusual response to tissue injury. It is distinguishable from pyogenic granuloma and peripheral ossifying fibroma only on the basis of its unique histomorphology [18, 19].

Peripheral odontogenic fibroma (WHO type) must be considered in the differential diagnosis of dome-shaped or nodular, nonulcerated growths on the gingiva like PGCG. Peripheral odontogenic fibroma is characterized by a fibrous or fibromyxomatous stroma containing varying numbers of islands and strands of odontogenic epithelium that is clearly distinguishable from PGCG histopathology [20].

The diagnosis was confirmed after histopathological analysis of the excised lesion. Histologically the PGCG can be differentiated from other reactive lesions mainly by the abundance of multinucleated giant cells [8, 11, 14], which is the same as central giant cell granuloma [18, 19] and only radiological evaluation can establish the distinction between central and peripheral forms of giant cell granulomas [14]. Radiographs are essential for confirming the oral mucosa origin of the giant cell lesion and refusing a central bony lesion with cortical perforation and soft tissue extension [16].

In the presented case, there was no radiographic evidence of bone involvement and no recurrence was observed.

Other entities need to be considered in differential diagnosis on the basis of histology, namely, brown tumor of hyperparathyroidism, cherubism, and aneurysmal bone cyst. This possibility should be considered, particularly if there are multiple lesions, if the same lesion recurs following appropriate surgical removal, and if there are radiographic alterations. [21, 22] Brown tumor of hyperparathyroidism can perforate the cervical region of the tooth and mimic GCG; alternatively, GCG may be the initial presentation of primary hyperparathyroidism or secondary hyperparathyroidism (generally due to renal insufficiency). Consideration of this disorder is particularly important in cases showing signs of hypercalcemia (renal calculi and neuromuscular, gastrointestinal, or psychiatric disorders), since patients with hyperparathyroidism have been reported to show increased blood levels of calcium, as well as parathormone and alkaline phosphates [23, 24]. However, the medical history of the patient was noncontributory.

Some investigators have suggested that history of trauma might be related to the development of PGCG [11, 15]. Although the patient did not relate any traumatic factor to the occurrence of the lesion, the fact of the left first premolar to be erupting improperly and partially reabsorbing the tooth root of primary canine and the patient's habit of “poking” the gingiva might have contributed to the development of the lesion.

The treatment of the PGCG involves the removal of irritating factors and, mainly, the surgical excision of the lesion, carefully curetting its edges and base, in order to reduce recurrences [10, 11, 25]. In the presented case, the patient was submitted to excisional biopsy of the lesion through its curettage with removal of the periosteum, periodontal ligament, and curettage of the teeth involved with vestibular access and there was no recurrence of the lesion. Surgical planning included the preservation of permanent lateral incisor and primary canine that, although involved, did not show increased mobility.

Early detection of the PGCG results in a more conservative surgery with reduced risk for tooth and bone loss, important issues when treating pediatric patients. After four years of clinical and radiographic control, patient shows no signs of relapse or tissue defects.

Careful medical history followed by complete physical, imaginological, and histopathological examination is critical procedures in the diagnosis process, aiming for a correct treatment plan and thereby reducing the possibility of recurrence and morbidity for patients.
